# Radiation cognition, government trust and their potential influencing factors in residents within 30 km of Qinshan nuclear power plant

**DOI:** 10.3389/fpubh.2026.1867469

**Published:** 2026-07-22

**Authors:** Sijia Yang, Xiaoji Hao, Long Yuan, Jiadi Guo, Zhongjun Lai, Zhiqiang Xuan, Donghang Wang, Guijia Hu, Shunfei Yu, Cuiping Lei, Yiyao Cao

**Affiliations:** 1 Department of Occupational Health and Radiological Protection, Zhejiang Provincial Center for Disease Control and Prevention, Hangzhou, China; 2 National Institute for Radiological Protection, Chinese Center for Disease Control and Prevention, Beijing, China

**Keywords:** government trust, influencing factors, nuclear emergency evacuation and protection, nuclear radiation, Qinshan nuclear power plant, radiation cognition

## Abstract

**Background and objectives:**

Despite growing nuclear energy demand, public concerns over nuclear accidents persist, making residents’ perceptions and attitudes surrounding nuclear power plants particularly crucial. Therefore, this study aims to assess residents’ radiation-related knowledge and their trust in the government during nuclear accidents, and to analyze the potential influencing factors thereof.

**Methods:**

Participants were recruited via a stratified multi-stage random sampling approach from areas within 30 km of the Qinshan Nuclear Power Plant. A questionnaire survey was conducted to collect participants’ basic information, their knowledge of nuclear radiation (NR) and nuclear emergency evacuation and protection (NEEP), and their trust in the government regarding NEEP. Spearman’s rank correlation analysis was adopted to explore the correlations between participants’ subjective and objective cognition of radiation-related knowledge, as well as between the cognitive and behavioral dimensions of public trust. Multiple linear regression or logistic regression models were applied to identify potential influencing factors.

**Results:**

Approximately 20% of participants subjectively perceived good mastery of radiation knowledge, with a mean objective score of around 5 out of 8. Age, gender, education, and distance from the nuclear power plant were significantly associated with objective knowledge. Nearly 80% expressed trust in the government during nuclear emergencies, and trust was positively correlated with behavioral compliance. Specifically, greater trust in the government was associated with a stronger willingness to evacuate as arranged. Education, spatial distance from the nuclear power plant, concerns about discrimination after evacuation, worries about family members who cannot evacuate, and objective cognition of NEEP were found to be associated with public trust.

**Conclusion:**

Residents within a 30-km radius of the Qinshan Nuclear Power Plant exhibited a generally positive level of cognition regarding radiation-related knowledge and trust in the government during NEEP, while significant room for improvement remains. Given the critical role of public perception and trust in the sustainable development of nuclear energy, the potential influencing factors identified in this study may inform future longitudinal or interventional research aimed at enhancing public cognition and trust, thereby supporting the advancement of the nuclear energy industry.

## 1 Introduction

With the continuous growth in global energy demand and the urgent drive for energy structure transformation, nuclear energy, as a highly efficient and low-carbon energy source, has assumed an increasingly prominent strategic importance in the global energy system (1, 2). Currently, over 400 nuclear power reactors are in operation across 32 countries, with nuclear power generating about 10% of the global total electricity (3). This has provided crucial support for alleviating the environmental pressures and preventing premature deaths attributable to fossil fuel combustion (4).

Nevertheless, the advancement of nuclear energy has long been overshadowed by public anxieties regarding the risks of nuclear radiation (NR). Notably, catastrophic nuclear safety incidents in history, including the Chernobyl Nuclear Disaster of 1986 and the Fukushima Nuclear Accident of 2011, have not only inflicted substantial human casualties, property damage, and ecological degradation, but also precipitated a worldwide confidence crisis in the safety of nuclear technologies, the ramifications of which endure to the present day (5, 6).

As an invisible source of risk, NR is characterized by its technicality and complexity, which often lead to biases in the perception of it. Existing studies have indicated that the general public has a relatively low level of knowledge about NR, but they tend to perceive a high risk of radiation (7, 8). Such biases not only impede the sound development of the nuclear energy industry but also may induce irrational decision-making among the public during nuclear emergency evacuation and protection (NEEP), thereby exacerbating the harm caused by nuclear accidents (9, 10). It is therefore essential for the public to develop a correct understanding of radiation-related knowledge, which enables them to view it scientifically and avoid blind panic.

Evacuation plays a critical role in safeguarding public lives during nuclear accident emergency responses, and the level of public trust in governments and official institutions directly determines the efficiency of implementation (11, 12). Thus, clarifying public awareness of radiation-related knowledge and their trust in the government within the context of NEEP, as well as analyzing the influencing factors, holds significant theoretical and practical value for enhancing nuclear accident emergency management capabilities, protecting public safety, and maintaining social stability.

As a major nuclear energy country, China ranks among the world’s top tier in nuclear power generation (13). Qinshan Nuclear Power Plant, widely recognized as the birthplace of China’s nuclear power industry, is located in Haiyan County, Zhejiang Province, and was completed and put into operation in 1991 (14). Qinshan Nuclear Power Plant was selected for several reasons. Its long operation guarantees reliable resident survey data. Its pressurized water reactors and surrounding demographic characteristics including mixed urban–rural and moderate population density, are representative of many coastal nuclear sites in China. Furthermore, available residential registration data enabled a robust sampling frame. In the present study, an investigation was conducted among residents living in the vicinity of Qinshan Nuclear Power Plant to analyze their cognitive and behavioral patterns regarding NR and NEEP, and the potential influencing factors.

## 2 Materials and methods

### 2.1 Study design

To investigate the public awareness of NR and NEEP, as well as their trust in the government, this study employed a stratified multi-stage random sampling method in 2024. Taking Qinshan Nuclear Power Plant as the center, the surrounding areas were divided into three concentric zones: within 10 km, 10–20 km, and 20–30 km from the plant, where a cross-sectional survey was conducted among the residents. This categorization simplified the sampling stratification recommended in the Chinese national standard for health surveys around nuclear power plants (WS/T 440–2014), which specifies the area into 10 categories. The practical simplification for primary analysis is consistent with a previous study (15), a classification that allows for comparability with existing literature.

The sample size calculation was based on the expected incidence rate in simple random sampling, and the formula employed is as follows:


$$n=\frac{Z^{2}\times p\times (1-p)}{d^{2}}$$

A prior study on NR-related awareness among residents near the nuclear power plant indicated that the public’s radiation awareness rate stood at approximately 40% (15). Therefore, the *p*-value in the formula was set at 0.4, and the permissible error *d* was 0.1 × *p* = 0.04. A *Z*-value of 1.96 was adopted for the 95% confidence level, yielding a calculated sample size (*n*) of 576. Considering the multi-stage stratified sampling design (design effect = 1.1), the final total sample size was set at 630.

Within the three concentric strata delineated around the nuclear power plant, one town (representing rural areas) and one sub-district (representing urban areas) were randomly selected from each stratum, with residents randomly sampled. Residents within 30 km of the power plant were sampled from permanent resident registries and community household lists. We recruited participants door-to-door using systematic random sampling (random start with a fixed interval) based on a randomly ordered list. In each stratum, 210 questionnaires were distributed, with the requirement that respondents had resided in their current place of residence for at least 1 year and had no abnormalities in cognitive or expressive abilities.

### 2.2 Details of the questionnaire

This questionnaire was formulated by the experts of the National Institute for Radiological Protection. It had an overall Cronbach’s *α* coefficient of 0.806, indicating acceptable internal consistency. The Kaiser-Meyer-Olkin value was 0.855, and Bartlett’s sphericity test reached statistical significance (*p* < 0.001). Exploratory factor analysis showed that all items had rotated factor loadings above 0.5, suggesting good structural validity.

#### 2.2.1 Basic information

The questionnaire survey was administered via face-to-face interviews, and participants completed it in electronic form. The questionnaire first collected the participants’ basic information, including gender, age, educational background, and the distance between their place of residence and the nuclear power plant.

#### 2.2.2 Participants’ awareness of radiation-related knowledge

The survey then assessed participants’ knowledge of NR and NEEP using subjective self-perception (“*Do you think you have an understanding of NR/NEEP?*”) and objective knowledge (eight related questions for each domain). All objective questions and corresponding answers are provided in Supplementary Table 1. Each correct response earned one point, with incorrect or “I do not know” responses receiving zero. Additionally, a multiple-choice question, “*What are the main ways you obtain information about NR and NEEP?*” identified main sources of NR and NEEP information.

#### 2.2.3 Public trust in the government during NEEP

Subsequently, participants’ cognitive trust in the government and their intended behavioral responses during a nuclear accident were investigated. At the cognitive level, the questionnaire included the question “*In the event of a nuclear accident, do you believe the government can help residents evacuate safely?*” At the behavioral level, participants’ intentions were determined by their responses to “*If you learn of a serious nuclear accident at a nearby nuclear power plant, how would you evacuate?*” Furthermore, potential factors influencing public trust were examined, including concerns about post-evacuation discrimination following a nuclear accident, as well as safety concerns regarding family members and property.

Finally, the motivations underlying participants’ choices regarding NEEP were further explored. For participants who trusted government arrangements, the factors driving their decision were investigated using the question “*In a nuclear accident, what are the advantages of following government arrangements over self-evacuation?*” Their preferences for evacuation transportation were also examined with “*If you choose government-arranged evacuation, what mode of transportation would you prefer?*” For those who opted for self-evacuation, the primary reasons for their choice were analyzed through “*What is the main reason for choosing self-evacuation over following government arrangements?*” Meanwhile, their preferred self-evacuation destinations were surveyed via “*In the event of a nuclear accident, if you choose to self-evacuate, which location would you prefer?*” The question “*When preparing to evacuate, what would you do if your family members are hospitalized, in a nursing home for older adults, or at school (children)?*” was used to explore how participants would arrange evacuation for family members unable to evacuate independently. Furthermore, participants’ trust in official guidance and government decisions was examined in greater detail through a question regarding post-evacuation return to their original residence “*In the event of a nuclear accident, if you have been evacuated to another location for more than 6 months and the government announces that return to your original residence is permitted, where would you prefer to settle?*”

### 2.3 Quality control

Investigators of this survey, all from centers of disease prevention and control with medical backgrounds, received standardized pre-survey training. Questionnaires were signed by investigators and audited by reviewers for completeness, readability, and logical consistency. Daily meetings were held to address survey-related issues, implement quality control, and continuously optimize workflow. Questionnaire data were double-entered and cross-validated using EpiData 3.1 software.

### 2.4 Ethical approval and informed consent from participants

This study has been reviewed and approved by the Ethics Committee of the National Institute for Radiological Protection, Chinese Center for Disease Control and Prevention. The ethical review number is LLSC-NIRP 2024-002. Prior to the questionnaire survey, participants were required to sign an informed consent form, confirming their understanding of the study and voluntary participation.

### 2.5 Statistical analysis

In the present study, we treated the sample as a simple random sample because our primary goal is to test associations. Continuous variables were presented as mean ± standard deviation, and categorical variables as percentages. Spearman’s rank correlation analysis was used to examine the differences in participants’ subjective and objective cognition of radiation-related knowledge, as well as the correlation between the cognitive and behavioral dimensions of public trust in the government. Multivariate linear regression analysis was employed to assess the effects of various influencing factors on participants’ objective cognition of radiation-related knowledge, while logistic regression analysis was used to explore the associations between selected variables and public trust during a nuclear incident. Multicollinearity among independent variables was assessed using the variance inflation factor (VIF). The goodness-of-fit of the regression models was evaluated using appropriate indices. The proportional odds assumption was tested to justify the use of ordinal logistic regression. All statistical analyses were performed using IBM SPSS Statistics (Version 25.0), and all figures were generated with GraphPad Prism (Version 5.0). A two-tailed *p*-value < 0.05 was considered statistically significant.

## 3 Results

### 3.1 Characteristics of the participants

In the present study, a total of 601 participants submitted complete questionnaires, yielding an effective response rate of 95.4%. Such a high response rate minimizes the risk of non-response bias, as the proportion of non-respondents (4.6%) was too small to substantially affect the representativeness of the sample. Detailed demographic characteristics are presented in Table 1. The mean age was 45.77 ± 17.76 years. Since the correlation between cognitive level and age may not be linear, participants were stratified into four age groups: 18–29 (22.63%), 30–45 (32.61%), 46–60 (24.29%), and over 60 (20.47%). The gender ratio was balanced, with males accounting for 50.08% and females 49.92%. Regarding educational attainment, the proportion of participants increased with educational level, and those with education above junior high school constituted 64.22%. Within the three concentric strata centered on the nuclear power plant, participants were distributed relatively evenly, with respective proportions of 34.11%, 32.11%, and 33.78%. For nuclear emergency evacuation, 24.79% of participants expressed concerns about post-evacuation discrimination. In addition, 40.77% reported being completely worried about family members unable to evacuate, while nearly half (48.75%) had no concerns at all regarding the safety of their family property.

**Table 1:** General characteristics of the participants.

Variable	*n*	Mean ± standard deviation or percent
Age (years)	601	45.77 ± 17.76
18–29	136	22.63
30–45	196	32.61
46–60	146	24.29
>60	123	20.47
Gender	601	
Male	301	50.08
Female	300	49.92
Educational attainment	601	
No formal education	42	6.99
Primary school	73	12.15
Junior high school	100	16.64
Senior high school, technical school, and junior college	178	29.62
Bachelor’s degree or above	208	34.60^a^
Distance to the nuclear power plant	601	
<10 km	205	34.11
10–20 km	193	32.11
20–30 km	203	33.78
Concerns about discrimination after evacuation	601	
Yes	149	24.79
No	315	52.41
I do not know	137	22.80
Worry about family members who cannot evacuate	601	
Completely worried	245	40.77
Very worried	164	27.29
Moderate	117	19.47
Not very worried	44	7.32
Not worried at all	31	5.15 ^a^
Worry about the family property	601	
Completely worried	28	4.66
Very worried	48	7.99
Moderate	86	14.31
Not very worried	146	24.29
Not worried at all	293	48.75

a When the total percentage does not equal 100% after rounding, redistribute the difference to the last group of data. The upper section of the table contains the basic information of the participants, whereas the lower section outlines the potential influencing factors of their decision-making during nuclear accident evacuation.

### 3.2 Awareness of radiation-related knowledge

#### 3.2.1 Differences in subjective self-perception and objective cognition

Participants’ subjective cognition of NR was divided into five levels, ranging from “Ignorant” to “Completely knowledgeable.” The largest proportions rated themselves as “Not very knowledgeable” (27.62%) and “Moderate” (29.78%). Objective scores were calculated for each subjective level, and correlation analysis showed that objective scores increased with higher subjective cognition levels (*p* < 0.001), indicating that subjective and objective cognitive levels of NR were roughly consistent. Similarly, subjective cognition of NEEP was divided into five levels, with the highest proportions of respondents reporting “Ignorant” (26.12%) and “Not very knowledgeable” (30.95%). Consistent with the findings for NR, objective scores for NEEP also increased significantly with higher subjective cognition levels (*p* < 0.001), demonstrating consistency between subjective and objective cognition (Table 2).

**Table 2:** Differences in subjective versus objective cognition of participants regarding radiation-related knowledge.

Subjective cognition	*n*	Percent	Objective cognition score (Mean ± standard deviation)	*p* value^b^
NR	601		4.42 ± 2.34	
Ignorant	105	17.47	1.53 ± 1.76	
Not very knowledgeable	166	27.62	4.46 ± 2.18	
Moderate	179	29.78	5.24 ± 1.69	
Quite knowledgeable	107	17.80	5.62 ± 1.83	
Completely knowledgeable	44	7.33^a^	4.89 ± 1.92	<0.001
NEEP	601		4.73 ± 1.71	
Ignorant	157	26.12	3.80 ± 1.84	
Not very knowledgeable	186	30.95	4.73 ± 1.61	
Moderate	144	23.96	5.26 ± 1.47	
Quite knowledgeable	83	13.81	5.37 ± 1.29	
Completely knowledgeable	31	5.16	5.32 ± 1.54	<0.001

a When the total percentage does not equal 100% after rounding, redistribute the difference to the last group of data.

b *p* values were determined by Spearman’s rank correlation analysis. NR: nuclear radiation, NEEP: nuclear emergency evacuation and protection.

#### 3.2.2 Potential influencing factors of participants’ objective cognition

Multivariate linear regression analysis was conducted to identify potential factors influencing participants’ objective cognition of radiation-related knowledge (Table 3). In the regression model, the VIF values of all independent variables ranged from 1 to 3, suggesting no obvious multicollinearity. For NR, the model was statistically significant (*F* = 127.90, *p* < 0.001), and the adjusted *R*^2^ of 0.458 reflected moderate model fit. Regression results revealed that age, gender, education, and distance to the nuclear power plant were key determinants. Specifically, objective scores decreased with increasing age, with a statistically significant reduction observed in the >60 age group (*β* = −1.548, 95% CI: −2.165, −0.931). Females scored lower than males (*β* = −0.379, 95% CI: −0.646, −0.111), while educational attainment was positively associated with scores (*p* < 0.001). Additionally, participants residing within 10 km of the nuclear power plant obtained the highest scores (*p* < 0.001). The correlations between each variable and the objective score for NR-related knowledge are shown in Supplementary Table 2. For NEEP, the regression model was also statistically significant (*F* = 35.01, *p* < 0.001), with an adjusted *R*^2^ of 0.185, suggesting that the model was statistically valid but had relatively low explanatory power. No significant gender difference was found in objective scores. Scores followed an inverted U-shaped pattern with age, with the 46–60 age group scoring significantly higher than the 18–29 age group (*β* = 0.453, 95% CI: 0.036, 0.871). Furthermore, scores were positively correlated with educational attainment (*p* < 0.001). Participants living 20–30 km from the nuclear power plant had significantly lower scores than those within 10 km (*β* = −0.517, 95% CI: −0.811, −0.222). The correlations between each variable and objective scores of NEEP-related knowledge are shown in Supplementary Table 3.

**Table 3:** Multivariate^a^ linear regression analysis of participants’ objective cognition regarding radiation-related knowledge.

Variable	NR β (95% CI)	NEEP β (95% CI)
Age (years)		
18–29	Referent	Referent
30–45	−0.038 (−0.403, 0.327)	0.273 (−0.059, 0.604)
46–60	−0.141 (−0.600, 0.319)	0.453 (0.036, 0.871)^*^
>60	−1.548 (−2.165, −0.931)^***^	−0.086 (−0.646, 0.474)
Gender		
Male	Referent	Referent
Female	−0.379 (−0.646, −0.111)^**^	0.109 (−0.133, 0.352)
Educational attainment		
No formal education	Referent	Referent
Primary school	1.540 (0.893, 2.188)^***^	1.218 (0.631, 1.806)^***^
Junior high school	1.872 (1.175, 2.568)^***^	1.563 (0.931, 2.195)^***^
Senior high school, technical school, and junior college	3.008 (2.269, 3.748)^***^	2.197 (1.526, 2.869)^***^
Bachelor’s degree or above	3.812 (3.042, 4.582)^***^	2.484 (1.785, 3.183)^***^
Distance to the nuclear power plant		
<10 km	Referent	Referent
10–20 km	−0.814 (−1.144, −0.485)^***^	−0.041 (−0.340, 0.258)
20–30 km	−0.478 (−0.802, −0.153)^**^	−0.517 (−0.811, −0.222)^**^

a Adjusted for age, gender, educational attainment and distance to the nuclear power plant. NR, nuclear radiation; NEEP, nuclear emergency evacuation and protection; β, regression coefficient; CI, confidence interval. ^*^p < 0.05, ^**^p < 0.01, ^***^p < 0.001. *n* = 601.

**Figure 1:**
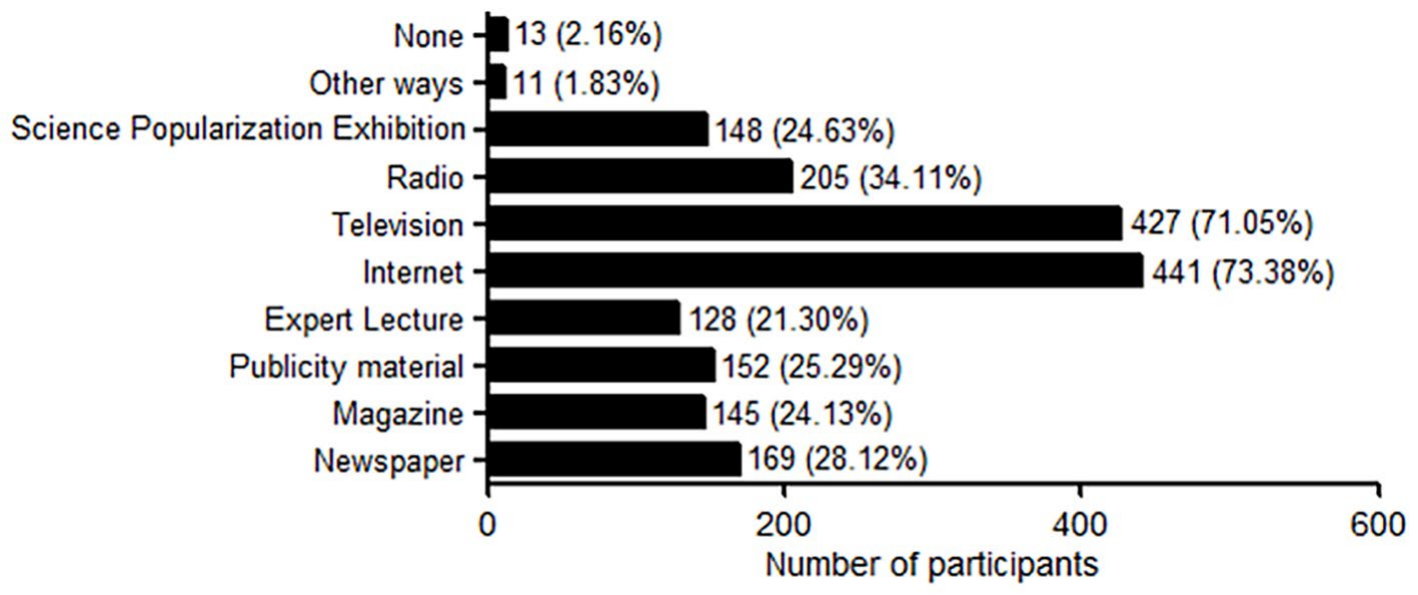
Key ways participants acquire radiation-related knowledge. Parenthetical percentages denote the proportion of respondents who selected each option out of the total (*n* = 601).

Next, the key ways through which participants acquired radiation-related knowledge were analyzed. As shown in Figure 1, most of the 601 participants obtained their knowledge from the Internet (73.38%) and television (71.05%), followed by radio (34.11%), newspapers (28.12%), publicity materials (25.29%), science popularization exhibitions (24.63%), magazines (24.13%), and expert lectures (21.30%).

### 3.3 Public trust in the government during NEEP

#### 3.3.1 The correlation between participants’ cognitive trust and their intended behavioral responses

Cognitive-level public trust in the government was categorized into five grades, with 80.03% of the 601 participants believing that the government could assist them in safe evacuation following a nuclear accident. To analyze the consistency between cognitive and behavioral trust levels, Spearman’s rank correlation analysis was employed (Table 4). The results indicated that higher cognitive trust was associated with a significantly greater willingness to evacuate in accordance with government arrangements (*p* < 0.001).

**Table 4:** Correlation between cognitive and behavioral level of public trust in the government.

Cognitive level	*n*	Percent^a^	Behavioral level *n* (percent)^b^		*p* value^c^
			Self evacuation	Arranged by the government	
Completely unconvinced	26	4.33	8 (34.78)	15 (65.22)	
Not very convinced	22	3.66	11 (57.89)	8 (42.11)	
Moderate	72	11.98	31 (44.93)	38 (55.07)	
Quite convinced	183	30.45	33 (18.54)	145 (81.46)	
Completely convinced	298	49.58	18 (6.12)	276 (93.88)	<0.001

a Percentage relative to the total of 601 participants at the cognitive level.

b Excluding 18 “I do not know” responses, a total of 583 participants were included at the behavioral level. “Percent” refers to the percentage relative to the participants in each cognitive level.

c *p* values were determined by Spearman’s rank correlation analysis.

**Figure 2:**
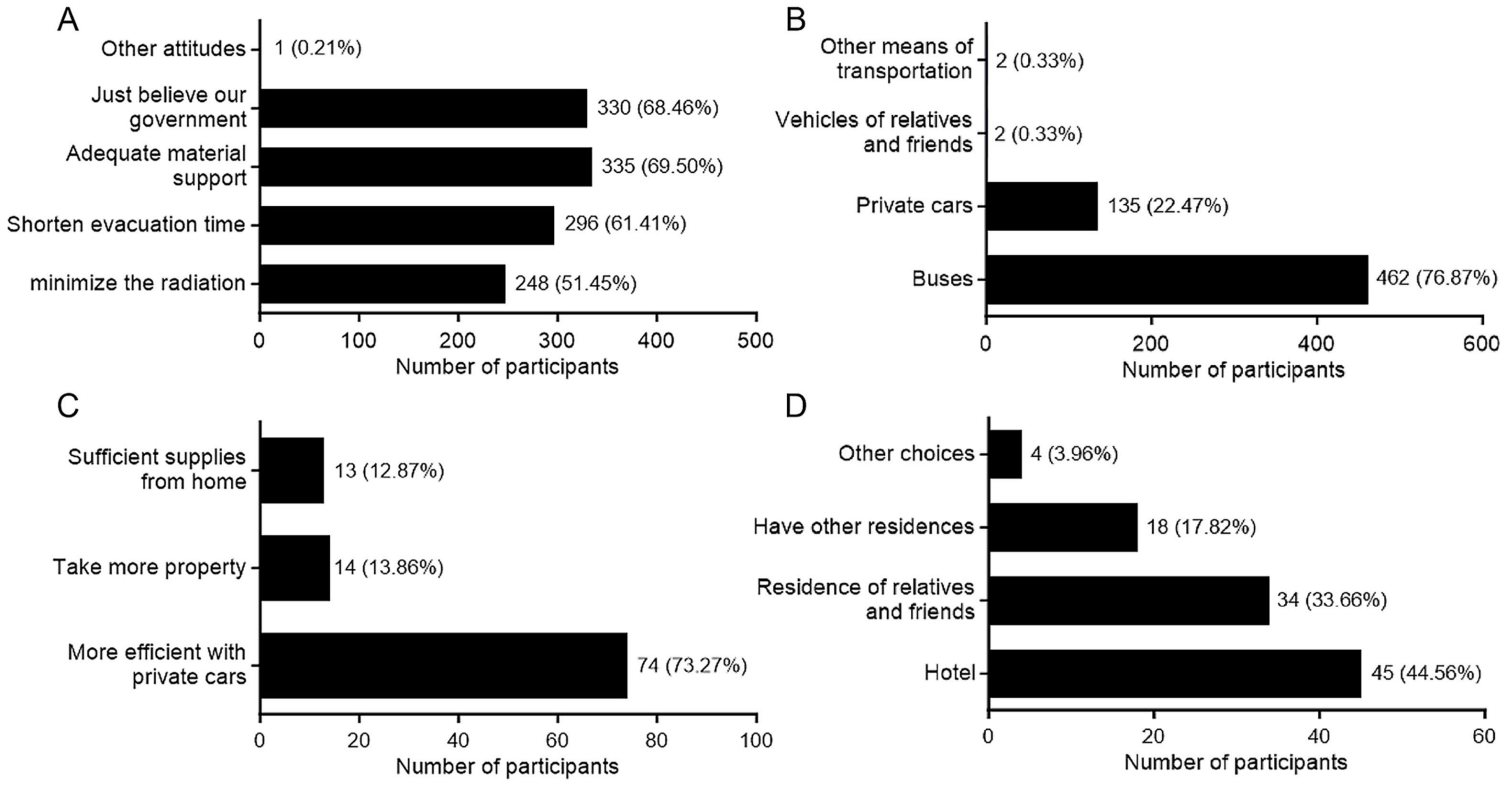
Participants’ views on evacuation after a nuclear accident. **(A)** Responses to “*In a nuclear accident, what are the advantages of following government arrangements over self-evacuation?*” (multiple-choice, *n* = 482). **(B)** Responses to “*If you choose government-arranged evacuation, what mode of transportation would you prefer?*” (single-choice, *n* = 601). **(C)** Responses to “*What is the main reason for choosing self-evacuation over following government arrangements?*” (single-choice, *n* = 101). **(D)** Responses to “*In the event of a nuclear accident, if you choose to self-evacuate, which location would you prefer?*” (single-choice, *n* = 101).

Among the 482 participants who opted for government-organized evacuation, 69.50% believed the government could provide adequate material support, 68.46% expressed direct trust in the government, 61.41% considered the government capable of shortening evacuation time, and 51.45% thought that this choice would minimize radiation exposure (Figure 2A). For evacuation transportation preferences, the majority of participants favored government-arranged buses (76.87%), while 22.47% chose private cars (Figure 2B). Among the 101 participants who chose self-evacuation, 73.27% deemed private vehicle evacuation more efficient (Figure 2C). For self-evacuation destinations, the top two choices were hotels (44.56%) and relatives’ or friends’ residences (33.66%) (Figure 2D).

#### 3.3.2 Potential influencing factors of public trust in the government

Table 5 and Supplementary Table 4 present the potential influencing factors of public trust at cognitive and behavioral levels using logistic regression analysis. The regression model in Table 5 included nine factors, whereas that in Supplementary Table 4 included only four factors, including gender, age, education, and distance to the nuclear power plant. Multicollinearity diagnostics revealed that all VIF values were approximately 2, confirming the absence of multicollinearity among the independent variables. As shown in Supplementary Table 4, participants residing 20–30 km from the nuclear power plant exhibited significantly lower trust at both the cognitive (OR = 0.592, 95% CI: 0.410, 0.854) and behavioral (OR = 0.488, 95% CI: 0.291, 0.818) levels compared with those living within 10 km.

**Table 5:** Multivariate^a^ logistic regression analysis of public trust in the government.

Variable	Cognitive level^b^ OR (95% CI)	Behavioral level^c^ OR (95% CI)
Age (years)		
18–29	Referent	Referent
30–45	0.654 (0.417, 1.027)	0.878 (0.455, 1.695)
46–60	0.828 (0.467, 1.469)	0.723 (0.321, 1.628)
>60	0.748 (0.339, 1.650)	0.356 (0.115, 1.095)
Gender		
Male	Referent	Referent
Female	1.161 (0.836, 1.611)	1.124 (0.699, 1.810)
Educational attainment		
No formal education	Referent	Referent
Primary school	0.473 (0.208, 1.074)	0.846 (0.256, 2.796)
Junior high school	0.390 (0.157, 0.970)^*^	0.620 (0.167, 2.308)
Senior high school, technical school, and junior college	0.348 (0.131, 0.928)^*^	0.382 (0.093, 1.560)
Bachelor’s degree or above	0.339 (0.120, 0.959)^*^	0.381 (0.085, 1.718)
Distance to the nuclear power plant		
<10 km	Referent	Referent
10–20 km	1.735 (1.130, 2.663)^*^	1.527 (0.791, 2.947)
20–30 km	0.727 (0.484, 1.092)	0.600 (0.335, 1.075)
Concerns about discrimination after evacuation		
Yes	Referent	Referent
No	2.409 (1.615, 3.595)^***^	3.879 (2.215, 6.791)^***^
I do not know	1.694 (1.026, 2.798)^*^	2.711 (1.347, 5.459)^**^
Worry about family members who cannot evacuate		
Completely worried	Referent	Referent
Very worried	0.393 (0.254, 0.610)^***^	1.089 (0.561, 2.114)
Moderate	0.275 (0.171, 0.443)^***^	0.687 (0.349, 1.350)
Not very worried	0.174 (0.091, 0.331)^***^	0.404 (0.172, 0.948)^*^
Not worried at all	0.112 (0.054, 0.233)^***^	0.284 (0.112, 0.721)^**^
Worry about the family property		
Completely worried	Referent	Referent
Very worried	0.793 (0.290, 2.168)	0.261 (0.028, 2.465)
Moderate	0.595 (0.231, 1.527)	0.350 (0.040, 3.080)
Not very worried	0.936 (0.378, 2.314)	0.445 (0.052, 3.809)
Not worried at all	1.367 (0.582, 3.213)	0.367 (0.045, 2.977)
Objective cognition of NR	0.987 (0.888, 1.096)	0.933 (0.800, 1.088)
Objective cognition of NEEP	1.129 (1.001, 1.274)^*^	1.251 (1.047, 1.495)^*^

a Adjusted for age, gender, education, distance to the nuclear power plant, various concerns (discrimination, family members, and property), objective knowledge cognition.

b Results of government trust from the cognitive level were obtained by ordinal logistic regression analysis. *n* = 601.

c Results of government trust from the behavioral level were obtained by binary logistic regression analysis. *n* = 583.

OR, odd ratio; CI, confidence interval; NR, nuclear radiation; NEEP, nuclear emergency evacuation and protection. ^*^p < 0.05, ^**^ *p* < 0.01, ^***^ *p* < 0.001.

At the cognitive level (Table 5), the model was statistically significant (likelihood ratio *χ*^2^ = 150.75, *p* < 0.001), with a Nagelkerke *R*^2^ of 0.243. The test of parallel lines confirmed the proportional odds assumption (*χ*^2^ = 12.40, *p* > 0.05), justifying the use of ordinal logistic regression. Participants with an education level of junior high school, senior high school, or bachelor’s degree or above exhibited lower trust compared to those with no formal education (*p* < 0.05). Residents living 10–20 km from the nuclear power plant exhibited higher trust in the government than those in closer proximity (OR = 1.735, 95% CI: 1.130, 2.663). Participants responding with no concerns about post-evacuation discrimination (OR = 2.409, 95% CI: 1.615, 3.595) or “I do not know” (OR = 1.694, 95% CI: 1.026, 2.798) had a higher trust level than those with such concerns. Participants with low concern about family members unable to evacuate had lower trust in the government than those completely worried (*p* < 0.001). Additionally, NEEP scores were positively associated with public trust in the government (OR = 1.129, 95% CI: 1.001, 1.274). Correlations between each variable and cognitive-level public trust are presented in Supplementary Table 5.

At the behavioral level (Table 5), the binary logistic regression model was statistically significant (Omnibus *χ*^2^ = 69.18, *p* < 0.001), with a Nagelkerke *R*^2^ of 0.186 and an overall predictive accuracy of 82.85% from the classification table. Compared with participants worried about post-evacuation discrimination, those with no such concerns (OR = 3.879, 95% CI: 2.215, 6.791) and those who responded “I do not know” (OR = 2.711, 95% CI: 1.347, 5.459) were more willing to follow government evacuation instructions. However, participants who reported “not very worried” (OR = 0.404, 95% CI: 0.172, 0.948) or “not worried at all” (OR = 0.284, 95% CI: 0.112, 0.721) about family members being unable to evacuate were less willing to do so than those who were completely worried. In addition, participants with higher objective NEEP scores were more willing to evacuate in accordance with government instructions (OR = 1.251, 95% CI: 1.047, 1.495). Correlations between each variable and public trust at the behavioral level are shown in Supplementary Table 6.

**Figure 3:**
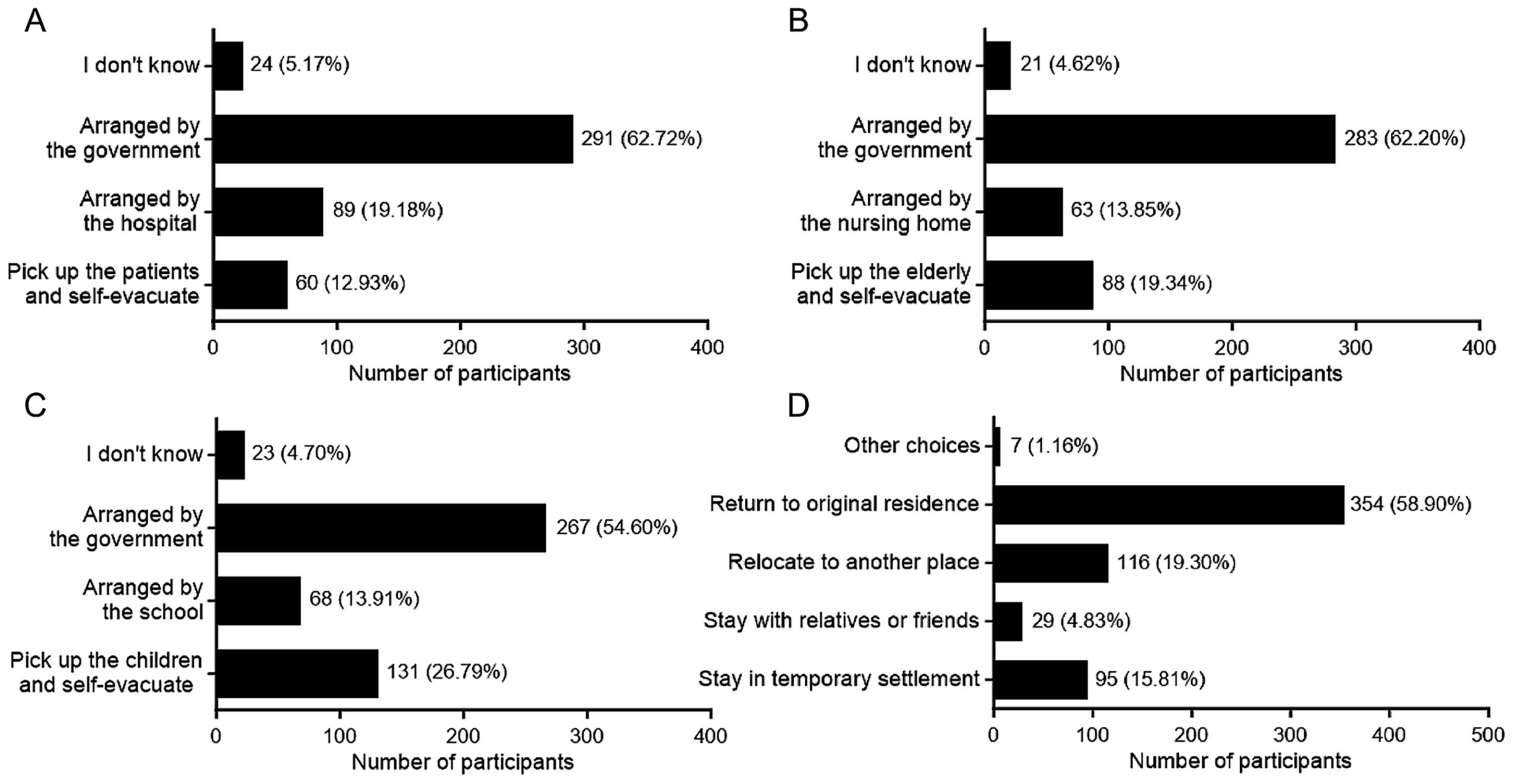
Participants’ arrangements for evacuating their family members in the event of a nuclear accident, and their choices of post-evacuation destinations. **(A–C)** Responses to “*When preparing to evacuate, what would you do if your family members are hospitalized* **(A)***, in a nursing home for older adults* **(B)***, or at school (children)* **(C)**?” (*n* = 601). **(D)** Responses to “*In the event of a nuclear accident, if you have been evacuated to another location for more than 6 months and the government announces that return to your original residence is permitted, where would you prefer to settle?*” (*n* = 601).

Given that concerns about family members unable to evacuate independently were significantly associated with participants’ evacuation decisions, we conducted a more detailed survey on their arrangements for family members, including patients in hospitals (Figure 3A), older adults in nursing homes (Figure 3B), and children at school (Figure 3C). The results showed that most participants opted for unified evacuation arrangements organized by the government or official agencies, with percentages of 81.90%, 76.05%, and 68.51% for the three scenarios, respectively. Conversely, 12.93%, 19.34%, and 26.79%, respectively, chose to evacuate with family members on their own (Figures 3A–C). Furthermore, a survey on post-evacuation destination choices revealed that 58.90% of the respondents would return to their original residences, while 19.30% opted to relocate to another place and 15.81% chose to stay in temporary settlements (Figure 3D).

## 4 Discussion

In the present study, four main results emerged. Firstly, participants’ cognition regarding radiation-related knowledge was statistically consistent between subjective self-perception and objective knowledge. Secondly, factors including age, gender, educational attainment, and distance to the nuclear power plant were associated with objective knowledge. Thirdly, participants’ cognitive trust in the government and their intended behavioral responses regarding NEEP were positively correlated. Finally, education, distance to the nuclear power plant, concerns about discrimination after evacuation, worries about family members who cannot evacuate, and objective cognition of NEEP were significantly associated with public trust in the government.

According to our findings, participants’ subjective self-perception of radiation-related knowledge was statistically consistent with their objective knowledge. This suggests that individuals can assess radiation risks based on their actual knowledge level, neither underestimating such risks nor succumbing to blind panic (16). The observed consistency carries practical value for large-scale risk communication. It implies that residents’ self-rated understanding may serve as a reliable proxy for actual knowledge levels in routine community surveys, enabling the rapid identification of knowledge-poor subgroups without administering extensive knowledge tests. Interestingly, participants who self-reported as “completely knowledgeable” scored slightly lower than those who rated themselves as “quite knowledgeable.” This phenomenon can be explained by the Dunning-Kruger effect in psychology (17), a cognitive bias where individuals overestimate their competence. Moreover, in the 8-point objective knowledge assessment, even among participants who claimed to have a good understanding of radiation-related knowledge, the average score was only around 5, indicating a general need for enhanced public knowledge. An accurate grasp of knowledge pertaining to NR and NEEP constitutes a critical prerequisite for the sustainable development of nuclear energy (18–20). Therefore, exploring the potential factors influencing public understanding and formulating effective improvement strategies are of great practical significance.

The current analysis identified age, gender, education, and distance from nuclear power plants as key factors that associated with objective cognition of NR and NEEP. Limited access to relevant information and generally low educational attainment among participants aged over 60 may partially account for their relatively poor cognition of NR (21). In contrast, participants aged 46–60 achieved higher objective scores in NEEP than younger participants. This may be due to the fact that their formal education coincided with the critical take-off stage of China’s nuclear power industry, when nuclear energy education was widely promoted (22). Females scored lower than males in NR knowledge assessments. One possible interpretation is that males may have greater access to radiation-related knowledge through occupational channels (23), while females may exhibit a higher level of fear toward radiation risks, which could be associated with cognitive avoidance behaviors (24). Cognition of NR and NEEP showed a positive correlation with educational attainment, and those living more than 10 km from nuclear power plants had significantly lower scores than those living within 10 km. Consistent with the findings of previous studies (21, 25, 26), this indicates that both educational attainment and the spatial distance from participants’ residential areas to nuclear power plants may serve as important correlates of radiation-related knowledge. Based on the aforementioned factors and primary channels for accessing radiation-related knowledge, older adult and women with both lower knowledge scores and distinct information access patterns may benefit most from community-based, face-to-face educational sessions, whereas Internet and television campaigns can efficiently reach the broader population. Moreover, the distance-related difference suggests that geographically differentiated messaging may be warranted. Specifically, residents in closer zones, who face higher objective risk, may require more frequent and detailed updates, while those farther away could be served through general awareness campaigns. This tiered approach optimizes communication resources without compromising overall preparedness.

After a nuclear accident, evacuation is a primary emergency measure, and effective governmental organization is key to safeguarding lives and minimizing accident-related losses (18). More than 80% of the participants reported being “quite convinced” or “completely convinced” in the government, reflecting the remarkable effectiveness of prior nuclear emergency risk communication and outreach initiatives. In evacuation implementation, public trust is a core factor (27). Trust represents a cognitive attitude, while compliance reflects a behavioral measure (28). We thus analyzed the correlation between participants’ cognitive trust in the government and their intended behavioral choices regarding NEEP, to clarify the relationship between public trust and behavioral compliance. Our findings revealed a consistency between cognitive trust and behavioral choices, suggesting that subjective trust is closely linked to behavioral intentions Among participants who opted to follow government arrangements during a nuclear accident evacuation, 68.46% made this choice primarily due to their trust in government, and over half believed that government-organized orderly evacuation would guarantee sufficient material support, shorten evacuation time, and minimize radiation exposure. The findings support the aforementioned trust-behavior compliance model. In contrast, the majority of participants who chose self-evacuation believed this approach could enhance evacuation efficiency by utilizing private vehicles. Regarding evacuation destination, the largest proportion selected hotels or relatives’ and friends’ residences. Our findings reinforce that trust is not merely a psychological construct but a behavioral driver with measurable consequences. For NEEP, it implies that investments in trust-building yield tangible returns in the form of higher compliance with evacuation orders. Importantly, addressing the specific concerns that drive self-evacuation could further strengthen this trust-behavior linkage, converting cognitive confidence into cooperative action.

Finally, we identified several factors associated with participants’ cognitive trust in the government and their intended behaviors, including education, distance to the nuclear power plant, concerns about post-evacuation discrimination, worries about non-evacuable family members, and objective knowledge of NEEP. Educational attainment was negatively correlated with trust level, consistent with findings from a prior study (29). This may be explained by stronger critical thinking and information-seeking skills among more educated individuals. Participants living 10–20 km from the nuclear power plant exhibited higher cognitive trust in the government than those within 10 km, a difference likely reflecting heightened anxiety among individuals residing closer to the plant (30, 31). Our results also revealed that public concerns about discrimination after evacuation were associated with their trust, aligning with evidence that psychological problems are common after major nuclear accidents (32–34) and that distrust in government is linked to poor mental health following the Fukushima disaster (35). Therefore, public anxiety about discrimination and its resulting psychological issues deserve close attention. Another notable factor was concern for vulnerable family members, including older adults, patients, and children. These individuals are physically and mentally fragile, and evacuation can severely impact their health. During the Fukushima accident, inadequate advance planning, together with a lack of dedicated transportation, support systems, and residential care facilities, led to many preventable fatalities among hospitalized patients and care home residents (36). Hence, future emergency responses must urgently address the needs of this population. The positive correlation between objective knowledge of NEEP and trust in the government suggests that knowledge may play a role in trust. Consistent with another study (37), efforts to enhance public knowledge may be beneficial for building trust in effective emergency response. Associations identified here have practical implications for intervention design, suggesting that trust-building efforts may be multidimensional. First, addressing post-evacuation discrimination concerns requires explicit policy assurances. Second, planning for vulnerable family members through dedicated transportation, medical support, and accessible shelters alleviates a key source of public anxiety that undermines trust. Third, the positive NEEP knowledge-trust link suggests that educational interventions are necessary. An integrated strategy that combines factual education, psychological support, and logistical planning is therefore more likely to succeed than any single approach.

Unlike prior studies that separately explored radiation cognition or emergency evacuation intention, this study integrated residents’ subjective perception, objective knowledge level, governmental trust, and behavioral willingness within one analytical framework to comprehensively analyze the internal links. Additionally, this research further identified evacuation-related influencing factors, such as post-evacuation discrimination concerns and worries about non-evacuable family members, which are less discussed in the relevant literature. Moreover, we empirically identified the presence of the Dunning-Kruger effect in public radiation risk cognition and verified a positive linkage between governmental trust and compliant evacuation intention, illustrating the practical behavioral value of public trust in nuclear emergency scenarios.

Despite the above findings, this study has several limitations. First, the cross-sectional design precludes causal inferences. Thus, all associations reported herein should be interpreted as correlational, and future longitudinal studies are warranted to test causality. Second, the self-reported questionnaire may introduce social desirability and recall biases, and it mainly consists of relatively simple knowledge items, which may not fully capture the complexity of radiation literacy. Third, the investigation was carried out in a single region, limiting the generalizability of the findings. Fourth, only one town and one sub-district per distance zone were selected, which may confound distance effects with local characteristics. Fifth, residence duration, an important factor influencing residents’ perceptions of risk, trust, and familiarity with the power plant, is unavailable. Finally, information regarding general population distribution, sampling probabilities, and weighting is unavailable, so the statistical analysis treated the sample as a simple random sample without accounting for clustering or stratification, which may lead to underestimation of standard errors and an inflated Type I error rate. Therefore, the statistical significance should be interpreted with caution, and the results should be considered hypothesis-generating rather than confirmatory.

## 5 Conclusion

In summary, residents within a 30-km radius of the Qinshan Nuclear Power Plant demonstrated a generally positive level of radiation-related knowledge and trust in the government during NEEP, with considerable room for further improvement. Given that public cognition and attitudes have become a key factor constraining the sustainable development of the nuclear industry, especially against the backdrop of its rapid development, our findings are particularly timely. Specifically, the potential influencing factors of radiation cognition and government trust identified in this study highlight the need for future research that integrates risk management, health communication, and social psychology to design and evaluate strategies for enhancing public cognition and trust. Such efforts would help strengthen the social foundation for the sustainable development of the nuclear energy industry.

## Data Availability

The original contributions presented in the study are included in the article/Supplementary material, further inquiries can be directed to the corresponding authors.
