# Supplementary material for: Radiation cognition, government trust and their potential influencing factors in residents within 30 km of Qinshan nuclear power plant
Source: Front Public Health. 2026 Jul 22;14:1867469. doi: 10.3389/fpubh.2026.1867469 (PMC13437371; doi:10.3389/fpubh.2026.1867469)
Supplement: Supplementary file 1 [file Table_1.DOCX]

Supplementary Material

**Supplementary Table 1**. Objective questions on radiation-related knowledge and their corresponding answers in the questionnaire.

| **Category** | | **Question** | **Answer** |
| --- | --- | --- | --- |
|  | NR | 1. Is there no natural radiation in the environment we live in? | No |
|  |  | 2. Is uranium the main component of the nuclear fuel used in nuclear power plants? | Yes |
|  |  | 3. Will the reactor stop releasing heat immediately once a nuclear power plant is shut down? | No |
|  |  | 4. Will you not be exposed to radiation when undergoing a CT scan in the hospital? | No |
|  |  | 5. Will you not be exposed to radiation when having an ultrasound scan in the hospital? | Yes |
|  |  | 6. Is the solid waste generated by nuclear power plants, including spent nuclear fuel, non-radioactive? | No |
|  |  | 7. Is a nuclear power plant like a thermal power plant in that it needs to continuously add nuclear fuel to the reactor when generating electricity? | No |
|  |  | 8. Do you agree that all radioactive substances have the same half-life? | No |
|  | NEEP | 1. In the event of a nuclear accident, would you tend to take stable iodine? | Yes |
|  |  | 2. In the event of a nuclear accident, would you tend to take iodized salt if stable iodine is not available? | No |
|  |  | 3. In the event of a nuclear accident, would you tend to close the doors and windows and take shelter indoors? | Yes |
|  |  | 4. In the event of a nuclear accident, would you tend to leave the doors and windows open to keep the room ventilated? | No |
|  |  | 5. In the event of a nuclear accident, would you tend to follow the staff's instructions and evacuate to a safe location? | Yes |
|  |  | 6. In the event of a nuclear accident, would you tend to flee immediately to a distant place? | No |
|  |  | 7. In the event of a nuclear accident, would you tend to avoid local agricultural products if those supplied from other regions are available? | Yes |
|  |  | 8. In the event of a nuclear accident, would you tend to consume sealed packaged water or food if you have a choice? | Yes |

NR: nuclear radiation, NEEP: nuclear emergency evacuation and protection.

**Supplementary Table 2**. Correlations between each variable and objective cognition scores of NR-related knowledge.

| **Variable** | **Objective cognition score**  **(Mean ± standard deviation)** | ***P* value ^a^** |
| --- | --- | --- |
| Age (years) |  |  |
| 18-29 | 5.59 ± 1.68 |  |
| 30-45 | 5.32 ± 1.80 |  |
| 46-60 | 4.37 ± 2.13 |  |
| >60 | 1.76 ± 1.81 | <0.001 |
| Gender |  |  |
| Male | 4.60 ± 2.45 |  |
| Female | 4.24 ± 2.22 | 0.018 |
| Educational attainment |  |  |
| No formal education | 0.52 ± 0.77 |  |
| Primary school | 2.49 ± 2.02 |  |
| Junior high school | 3.53 ± 2.02 |  |
| Senior high school, technical school, and junior college | 4.93 ± 1.74 |  |
| Bachelor’s degree or above | 5.87 ± 1.64 | <0.001 |
| Distance to the nuclear power plant |  |  |
| <10 km | 4.88 ± 2.30 |  |
| 10-20 km | 3.97 ± 2.19 |  |
| 20-30 km | 4.38 ± 2.45 | 0.031 |

^a^ *P* values were determined by Spearman's rank correlation analysis. NR: nuclear radiation (n=601).

**Supplementary Table 3**. Correlations between each variable and objective cognition scores of NEEP-related knowledge.

| **Variable** | **Objective cognition score**  **(Mean ± standard deviation)** | ***P* value ^a^** |
| --- | --- | --- |
| Age (years) |  |  |
| 18-29 | 5.01 ± 1.50 |  |
| 30-45 | 5.17 ± 1.44 |  |
| 46-60 | 4.90 ± 1.48 |  |
| >60 | 3.52 ± 2.00 | <0.001 |
| Gender |  |  |
| Male | 4.68 ± 1.81 |  |
| Female | 4.79 ± 1.60 | 0.456 |
| Educational attainment |  |  |
| No formal education | 2.57 ± 2.10 |  |
| Primary school | 3.93 ± 1.83 |  |
| Junior high school | 4.47 ± 1.55 |  |
| Senior high school, technical school, and junior college | 5.04 ± 1.40 |  |
| Bachelor’s degree or above | 5.31 ± 1.39 | <0.001 |
| Distance to the nuclear power plant |  |  |
| <10 km | 4.93 ± 1.62 |  |
| 10-20 km | 4.86 ± 1.78 |  |
| 20-30 km | 4.41 ± 1.68 | 0.001 |

^a^ *P* values were determined by Spearman’s rank correlation analysis. NEEP: nuclear emergency evacuation and protection (n=601).

**Supplementary Table 4**. Multivariate ^a^ logistic regression analysis of public trust in the government.

| **Variable** | **Cognitive level ^b^**  **OR (95% CI)** | **Behavioral level ^c^**  **OR (95% CI)** |
| --- | --- | --- |
| Age (years) |  |  |
| 18-29 | Referent | Referent |
| 30-45 | 0.764 (0.503, 1.161) | 0.951 (0.519, 1.743) |
| 46-60 | 0.872 (0.515, 1.478) | 0.671 (0.324, 1.388) |
| >60 | 0.880 (0.433, 1.791) | 0.384 (0.144, 1.028) |
| Gender |  |  |
| Male | Referent | Referent |
| Female | 1.132 (0.833, 1.538) | 1.061 (0.684, 1.647) |
| Educational attainment |  |  |
| No formal education | Referent | Referent |
| Primary school | 0.811 (0.380, 1.732) | 1.032 (0.336, 3.171) |
| Junior high school | 0.853 (0.378, 1.925) | 0.895 (0.278, 2.888) |
| Senior high school, technical school, and junior college | 0.726 (0.307, 1.718) | 0.458 (0.135, 1.558) |
| Bachelor’s degree or above | 0.739 (0.302, 1.808) | 0.484 (0.135, 1.743) |
| Distance to the nuclear power plant |  |  |
| <10 km | Referent | Referent |
| 10-20 km | 1.362 (0.926, 2.003) | 1.429 (0.691, 2.257) |
| 20-30 km | 0.592 (0.410, 0.854)^**^ | 0.488 (0.291, 0.818)^**^ |

^a^ adjusted for age, gender, educational attainment, and distance to the nuclear power plant. ^b^ cognitive-level results for participants’ trust in the government were determined by ordinal logistic regression analysis (n=601). ^c^ behavioral-level results for participants’ trust in the government were determined by binary logistic regression analysis (n=583). OR: odd ratio, CI: confidence interval, ^**^ *P*<0.01.

**Supplementary Table 5**. Correlations between each variable and public trust at the cognitive level.

|  | **Cognitive level**  **n (percent)** | | | | |  |
| --- | --- | --- | --- | --- | --- | --- |
| **Variable** | **Completely unconvinced** | **Not very convinced** | **Moderate** | **Quite convinced** | **Completely convinced** | ***P* value ^a^** |
| Age (years) |  |  |  |  |  |  |
| 18-29 | 4 (2.94) | 1 (0.74) | 14 (10.29) | 51 (37.50) | 66 (48.53) |  |
| 30-45 | 12 (6.12) | 9 (4.59) | 28 (14.29) | 54 (27.55) | 93 (47.45) |  |
| 46-60 | 2 (1.37) | 5 (3.42) | 19 (13.01) | 50 (34.25) | 70 (47.95) |  |
| >60 | 8 (6.50) | 7 (5.69) | 11 (8.94) | 28 (22.77) | 69 (56.10) | 0.653 |
| Gender |  |  |  |  |  |  |
| Male | 14 (4.65) | 11 (3.65) | 36 (11.96) | 97 (32.23) | 143 (47.51) |  |
| Female | 12 (4.00) | 11 (3.67) | 36 (12.00) | 86 (28.66) | 155 (51.67) | 0.390 |
| Educational attainment |  |  |  |  |  |  |
| No formal education | 1 (2.38) | 3 (7.14) | 5 (11.90) | 8 (19.05) | 25 (59.52) |  |
| Primary school | 3 (4.11) | 4 (5.48) | 6 (8.22) | 23 (31.51) | 37 (50.68) |  |
| Junior high school | 4 (4.00) | 3 (3.00) | 10 (10.00) | 34 (34.00) | 49 (49.00) |  |
| Senior high school, technical school, and junior college | 9 (5.06) | 7 (3.93) | 28 (15.73) | 44 (24.72) | 90 (50.56) |  |
| Bachelor’s degree or above | 9 (4.33) | 5 (2.40) | 23 (11.06) | 74 (35.58) | 97 (46.63) | 0.422 |
| Distance to the nuclear power plant |  |  |  |  |  |  |
| <10 km | 9 (4.39) | 6 (2.93) | 20 (9.76) | 65 (31.71) | 105 (51.22) |  |
| 10-20 km | 8 (4.15) | 3 (1.55) | 20 (10.36) | 46 (23.83) | 116 (60.10) |  |
| 20-30 km | 9 (4.43) | 13 (6.40) | 32 (15.76) | 72 (35.47) | 77 (37.93) | 0.004 |
| Concerns about discrimination after evacuation |  |  |  |  |  |  |
| Yes | 10 (6.71) | 6 (4.03) | 28 (18.79) | 56 (37.58) | 49 (32.89) |  |
| No | 12 (3.81) | 9 (2.86) | 28 (8.89) | 73 (23.17) | 193 (61.27) |  |
| I don’t know | 4 (2.92) | 7 (5.11) | 16 (11.68) | 54 (39.42) | 56 (40.88) | 0.044 |
| Worry about family members who cannot evacuate |  |  |  |  |  |  |
| Completely worried | 9 (3.67) | 2 (0.82) | 16 (6.53) | 44 (17.96) | 174 (71.02) |  |
| Very worried | 2 (1.22) | 7 (4.27) | 17 (10.37) | 74 (45.12) | 64 (39.02) |  |
| Moderate | 6 (5.13) | 5 (4.27) | 25 (21.37) | 42 (35.90) | 39 (33.33) |  |
| Not very worried | 4 (9.09) | 5 (11.36) | 8 (18.18) | 16 (36.36) | 11 (25.00) |  |
| Not worried at all | 5 (16.13) | 3 (9.68) | 6 (19.35) | 7 (22.58) | 10 (32.26) | <0.001 |
| Worry about the family property |  |  |  |  |  |  |
| Completely worried | 3 (10.71) | 1 (3.57) | 1 (3.57) | 4 (14.29) | 19 (67.86) |  |
| Very worried | 0 (0.00) | 1 (2.08) | 4 (8.33) | 26 (54.17) | 17 (35.42) |  |
| Moderate | 3 (3.49) | 2 (2.33) | 23 (26.74) | 30 (34.88) | 28 (32.56) |  |
| Not very worried | 5 (3.42) | 7 (4.79) | 24 (16.44) | 54 (37.99) | 56 (38.36) |  |
| Not worried at all | 15 (5.12) | 11 (3.75) | 20 (6.83) | 69 (23.55) | 178 (60.75) | <0.001 |
| Objective cognition of NR | 26 (4.33) | 22 (3.66) | 72 (11.98) | 183 (30.45) | 298 (49.58) | 0.759 |
| Objective cognition of NEEP | 26 (4.33) | 22 (3.66) | 72 (11.98) | 183 (30.45) | 298 (49.58) | 0.023 |

^a^ *P* values were obtained by Spearman’s rank correlation analysis. NR: nuclear radiation, NEEP: nuclear emergency evacuation and protection. n=601.

**Supplementary Table 6**. Correlations between each variable and public trust at the behavioral level.

|  | **Behavioral level**  **n (percent)** | | |  |
| --- | --- | --- | --- | --- |
| **Variable** | **Self evacuation** | **Arranged by the government** | | ***P* value ^a^** |
| Age (years) |  |  |  | |
| 18-29 | 22 (16.18) | 114 (83.82) |  | |
| 30-45 | 32 (16.67) | 160 (83.33) |  | |
| 46-60 | 25 (17.48) | 118 (82.52) |  | |
| >60 | 22 (19.64) | 90 (80.36) | 0.476 | |
| Gender |  |  |  | |
| Male | 53 (18.21) | 238 (81.79) |  | |
| Female | 48 (16.44) | 244 (83.56) | 0.571 | |
| Educational attainment |  |  |  | |
| No formal education | 6 (17.65) | 28 (82.35) |  | |
| Primary school | 11 (15.94) | 58 (84.06) |  | |
| Junior high school | 15 (15.31) | 83 (84.69) |  | |
| Senior high school, technical school, and junior college | 34 (19.32) | 142 (80.68) |  | |
| Bachelor’s degree or above | 35 (16.99) | 171 (83.01) | 0.829 | |
| Distance to the nuclear power plant |  |  |  | |
| <10 km | 30 (14.85) | 172 (85.15) |  | |
| 10-20 km | 23 (12.11) | 167 (87.89) |  | |
| 20-30 km | 48 (25.13) | 143 (74.87) | 0.008 | |
| Concerns about discrimination after evacuation |  |  |  | |
| Yes | 45 (30.82) | 101 (69.18) |  | |
| No | 33 (10.58) | 279 (89.42) |  | |
| I don’t know | 23 (18.40) | 102 (81.60) | <0.001 | |
| Worry about family members who cannot evacuate |  |  |  | |
| Completely worried | 9 (3.67) | 2 (0.82) |  | |
| Very worried | 2 (1.22) | 7 (4.27) |  | |
| Moderate | 6 (5.13) | 5 (4.27) |  | |
| Not very worried | 4 (9.09) | 5 (11.36) |  | |
| Not worried at all | 5 (16.13) | 3 (9.68) | <0.001 | |
| Worry about the family property |  |  |  | |
| Completely worried | 30 (12.61) | 208 (87.39) |  | |
| Very worried | 22 (13.66) | 139 (86.34) |  | |
| Moderate | 24 (20.87) | 91 (79.13) |  | |
| Not very worried | 14 (35.00) | 26 (65.00) |  | |
| Not worried at all | 11 (37.93) | 18 (62.07) | 0.739 | |
| Objective cognition of NR | 101 (17.32) | 482 (82.68) | 0.770 | |
| Objective cognition of NEEP | 101 (17.32) | 482 (82.68) | 0.003 | |

^a^ *P* values of gender and concerns about discrimination were obtained by Pearson Chi-square test. *P* values of age, educational attainment, distance to the nuclear power plant, and worries about family members and property were obtained by Spearman’s rank correlation analysis. *P* values of objective cognition of NR and NEEP were obtained by Mann-Whitney U test. NR: nuclear radiation, NEEP: nuclear emergency evacuation and protection. n=583.
